# Genomic Analysis of wig-1 Pathways

**DOI:** 10.1371/journal.pone.0029429

**Published:** 2012-02-07

**Authors:** Yalda Sedaghat, Curt Mazur, Mahyar Sabripour, Gene Hung, Brett P. Monia

**Affiliations:** Department of Drug Discovery, Isis Pharmaceuticals Inc., Carlsbad, California, United States of America; University of Dayton, United States of America

## Abstract

**Background:**

Wig-1 is a transcription factor regulated by p53 that can interact with hnRNP A2/B1, RNA Helicase A, and dsRNAs, which plays an important role in RNA and protein stabilization. *in vitro* studies have shown that wig-1 binds p53 mRNA and stabilizes it by protecting it from deadenylation. Furthermore, p53 has been implicated as a causal factor in neurodegenerative diseases based in part on its selective regulatory function on gene expression, including genes which, in turn, also possess regulatory functions on gene expression. In this study we focused on the wig-1 transcription factor as a downstream p53 regulated gene and characterized the effects of wig-1 down regulation on gene expression in mouse liver and brain.

**Methods and Results:**

Antisense oligonucleotides (ASOs) were identified that specifically target mouse wig-1 mRNA and produce a dose-dependent reduction in wig-1 mRNA levels in cell culture. These wig-1 ASOs produced marked reductions in wig-1 levels in liver following intraperitoneal administration and in brain tissue following ASO administration through a single striatal bolus injection in FVB and BACHD mice. Wig-1 suppression was well tolerated and resulted in the reduction of mutant Htt protein levels in BACHD mouse brain but had no effect on normal Htt protein levels nor p53 mRNA or protein levels. Expression microarray analysis was employed to determine the effects of wig-1 suppression on genome-wide expression in mouse liver and brain. Reduction of wig-1 caused both down regulation and up regulation of several genes, and a number of wig-1 regulated genes were identified that potentially links wig-1 various signaling pathways and diseases.

**Conclusion:**

Antisense oligonucleotides can effectively reduce wig-1 levels in mouse liver and brain, which results in specific changes in gene expression for pathways relevant to both the nervous system and cancer.

## Introduction

*wig-1* is a p53-regulated gene (WT p53 induced gene 1; also known as PAG608 and ZMAT3) that was originally identified in a mouse cell line using a PCR-based differential display technique to find mRNAs induced by wild type p53 [1], [2]. The *wig-1* gene encodes a C_2_H_2_-type zinc finger protein that localizes mainly to the nucleus [3], [4]. The wig-1 structural features are shared with a small group of proteins, such as JAZ, that can positively regulate p53 transcriptional activity in a positive feedback manner [5], [6]. A rat homolog of *wig-1*, PAG608, was independently identified by Israeli *et al.* [2] and human *wig-1* has also been cloned and characterized [3]. Mouse wig-1 is highly homologous to the rat and human orthologs, and shares 97.9% and 87% amino acid sequence identity, respectively. Rat *wig-1* (PAG608) has weak pro-apoptotic activity when over-expressed in human tumor cells and human *wig-1* can suppress cell growth by 25–30% in a colony formation assay [2], [3].

wig-1 has also been shown to interact with heterogeneous nuclear ribonucleoprotein (hnRNP) A2/B1, RNA Helicase A (RHA), and dsRNA [4], [7], [8]. A relationship between wig-1 and p53 was also concluded in studies in which *wig-1* was suppressed by siRNA in vitro. It was shown that wig-1 binds to p53 mRNA in vitro and stabilizes it by protecting it from deadenylation. It was suggested that this effect is mediated by the U-rich region in the 3′ UTR of p53 mRNA [9]. Because p53 is involved in regulating cell death, it has the potential to play a significant role in the progression of neurodegenerative diseases including Huntington Disease (HD) where it has been found to affect phenotype in mouse models of HD [10]. Furthermore, a genetic interaction between the murine homologue of *huntingtin (htt)* and *p53* has also been reported to cause significant reductions in the severity of the HD phenotype in mice [11]. Moreover, recent in vitro and animal studies have shown that activation of p53 can promote huntingtin transcription and up-regulation of wild-type HTT protein, suggesting that *p53* and *htt* might interact functionally, and that changes in *p53* status may alter the HTT levels and, presumably, the HD phenotype [12].

The role of p53 in HD pathogenesis will likely involve different pathways, and it seems that targets of p53 activity might be responsible for different aspects of p53-related effects within neurodegenerative pathways. In this study we decided to focus on *wig-1* as a potential downstream target of p53 in neurodegenerative diseases by identifying genes that are potentially under regulation by *wig-1*. Microarray analysis was performed on the striatum of the HD mouse model, BACHD, treated locally with or without *wig-1* antisense oligonucleotides (ASOs), and on the liver following systemic treatment with wig-1 ASOs [13], [14], [15]. Our studies identify a number of neuronal and non-neuronal genes that are affected by *wig-1* expression in mouse brain and liver. Furthermore, genes found to be impacted by wig-1 suppression have been implicated in a wide range of critical pathways involved in neuronal function such as brain development and axon guidance, cancer, and mitochondrial function.

## Results

### Identification and characterization of a wig-1 antisense inhibitor *in vitro* and *in vivo*

**Figure 1:**
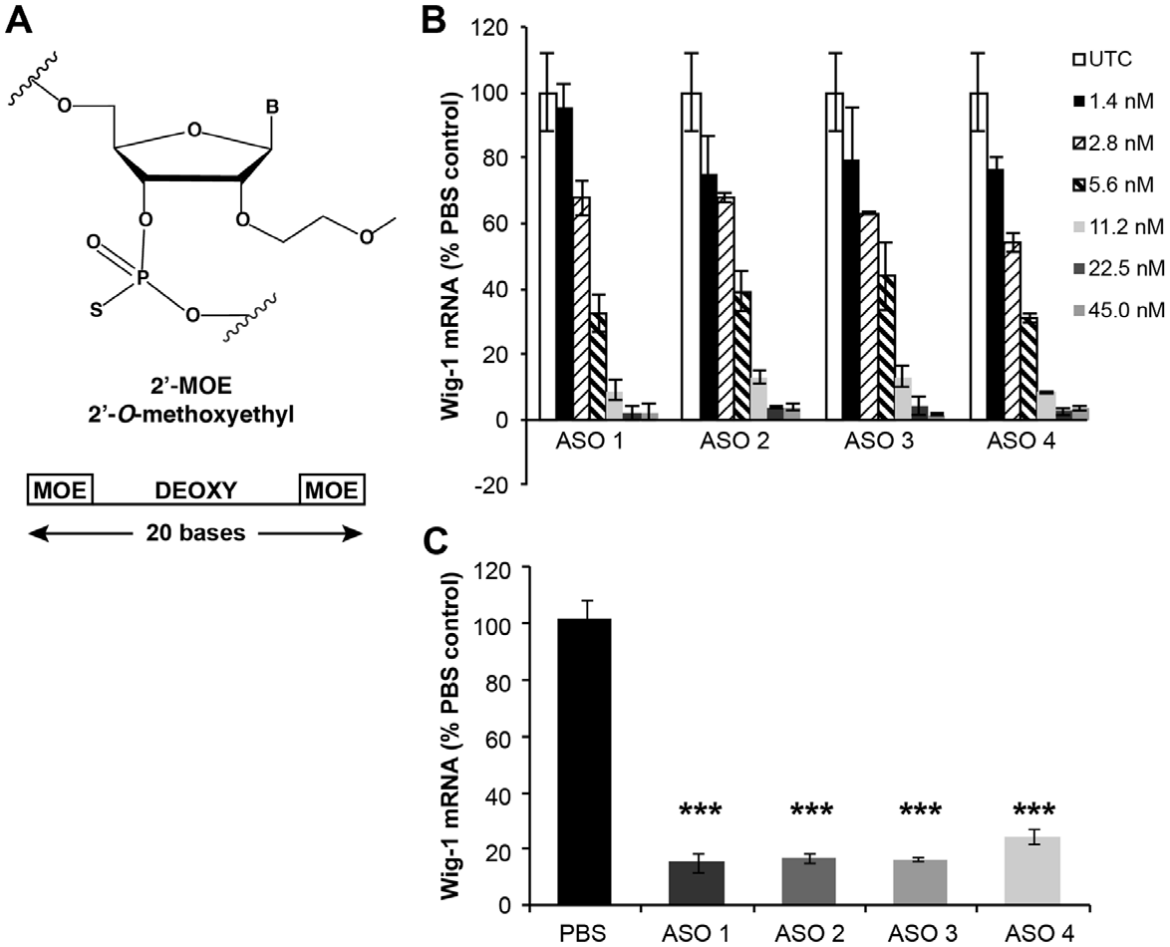
Mouse Wig-1 ASOs specifically reduce wig-1 mRNA levels in vitro and in vivo. A) oligonucleotides were 20 nucleotides in length and chemically modified with phosphorothioate in the backbone and 2′-*O*-methoxyethyl (MOE) on the wings with a central deoxy gap (“5-10-5” design). B) b.END cells were transfected with indicated concentration of wig-1 ASOs. RNA was extracted 24 hours after transfection and analyzed by RT-PCR to determine wig-1 mRNA levels. C) Male *BALB/c* mice were injected intraperitoneally with 4 different wig-1 ASOs at 100 mg/kg body weight per week or with saline for 4 weeks. Total RNA was prepared from liver, and used for real-time quantitative RT-PCR analysis to evaluate wig-1 mRNA levels. Data are expressed as means ± SEM (*n* = 4, ***p≤*0.01*).

To study the effects of wig-1 on htt and p53, we designed antisense oligonucleotides to specifically target mouse wig-1 mRNA. These chimeric ASOs contain ribonuclease H-sensitive stretches of 2′-deoxy residues flanked on both sides with a stretch of 2′-*O*-(2-methoxyethyl-ethyl) modifications, which increases RNA binding affinity and confers nuclease resistance. The central 2′-deoxy domain produces a substrate for endogenous RNase H enzymes once hybridized to the complementary target RNA, resulting in cleavage of the target RNA, thereby causing lowering of target protein levels [16] (Figure 1A). A series of ASOs were designed to bind within the coding region of the wig-1 mRNA sequence. Rapid-throughput screens were performed in the immortalized mouse brain endothelial cell line, bEND. Cells were transfected with ASOs, and harvested after 24 hours. The reduction of wig-1 expression was analyzed with real-time quantitative RT-PCR. Based on relative reduction in wig-1 mRNA levels, 4 ASOs were selected and further characterized in a dose-response screen. Wig-1 mRNA levels were significantly decreased in a dose-dependent manner with 90–95% reduction in wig-1 mRNA levels observed at a concentration of 45 nM (Figure 1B). These ASOs were further characterized in animals.

*In vivo* activity of wig-1 ASOs was characterized following intraperitoneal (IP) administration at 50 mg/kg or saline, twice a week for 4 weeks in BALB/c mice. Animals were euthanized 48 hours following the last dose. Serum transaminases, organ weights and organ histopathology were performed as a measure of overall tolerability. Liver samples were analyzed for changes in wig-1 mRNA levels and compared to saline treatment. All four ASOs reduced wig-1 mRNA levels by approximately 80% and ASO treatment was well tolerated (Figure 1C). One wig-1 ASO (ASO3) was selected for further studies.

### Characterization of wig-1 ASO activity in mouse brain

**Figure 2:**
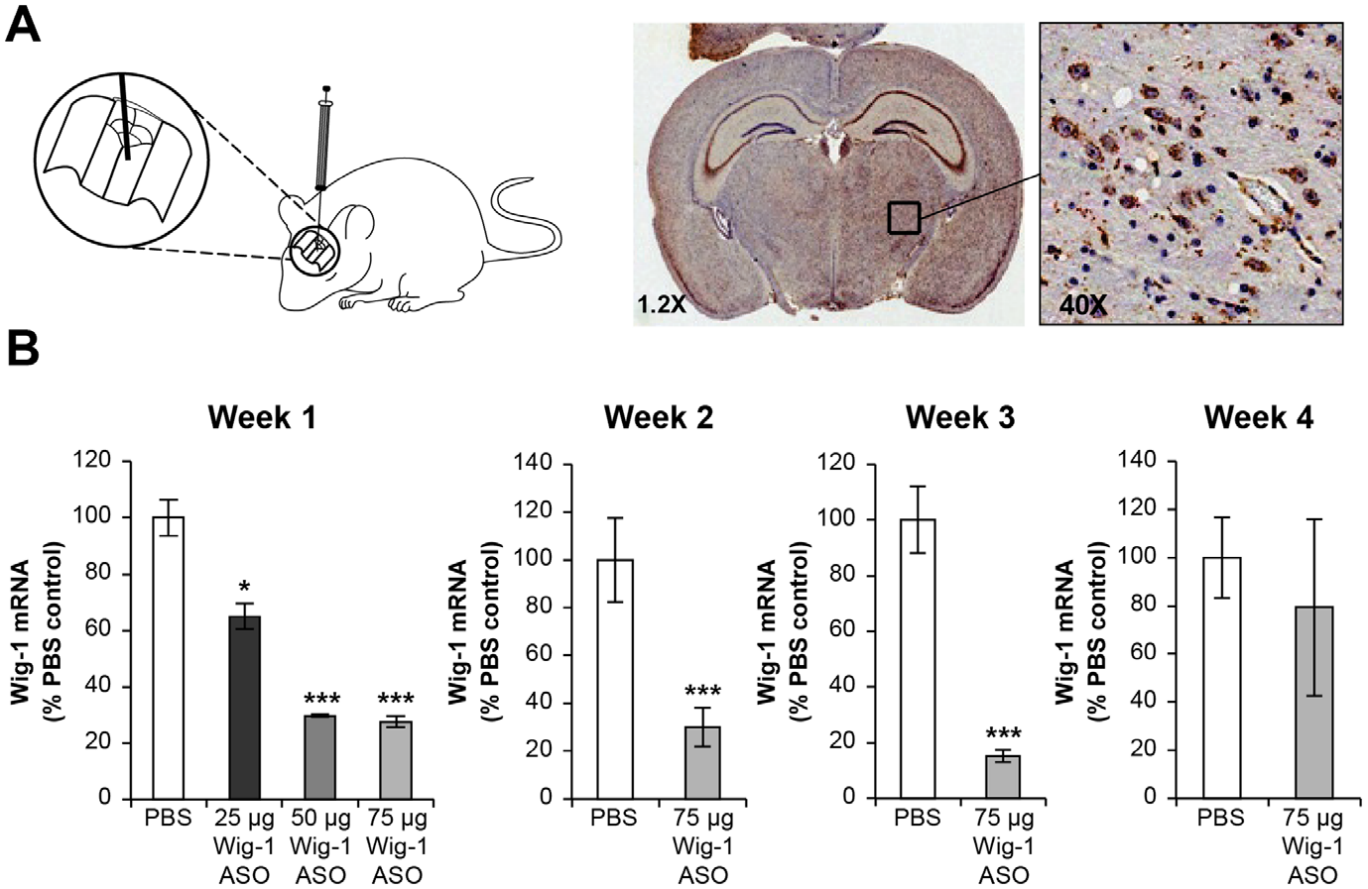
Dose-dependent reduction of wig-1 mRNA levels in mice brain following striatal bolus administration. A) Distribution of Wig-1 ASO was confirmed by staining with an antibody that recognizes oligonucleotides. At higher magnification ASO is visible in variuos cells including neurons, astrocytes. B) Male *FVB* mice received 75 µg single bolus injection of wig-1 ASO, or PBS in striatum. Animals were sacrificed after one, two, three, or four weeks. Striatum were harvested and total RNA was prepared from these sections, and used for real-time quantitative RT-PCR analysis to evaluate levels of wig-1 mRNA. Histological examination with hemotoxylin and eosin (H & E) staining did not show any remarkable abnormality in the brain of treated animals compare with control (data not shown). Data are expressed as means ± SEM (*n* = 4; **p≤0.05; ***p≤0.01).

Whole brain extracts of mice show relatively high expression levels of wig-1. To characterize the effects and the duration of action of the wig-1 ASO in mouse brain, we administered wig-1 ASO to FVB mice (the background strain for BACHD) (8-weeks-old) by a single bolus injection into the right striatum at 25, 50, and 75 µg and determined the effects on wig-1 mRNA and protein levels in brain tissue after one, two, three and four weeks post injection (Figure 2). Immunohistochemistry using an antibody that reacts with ASO confirmed ASO distribution to different regions of the brain as well as ASO uptake by different cell types (Figure 2A). Wig-1 ASO was taken up by both neuronal and glial cells, which is consistent with previous studies on ASO intrathecal administration in rats [17]. Immunostaining was present in sections of the brain, adjacent to the injection site as well as other regions, but staining decreased in intensity in a gradient as the distance increased from the injection site (Figure 2A). Hematoxylin and eosin staining of brain sections did not show any noticeable morphological abnormalities following ASO injection nor did any animals display abnormal behavior (data not shown). The magnitude of reduction of wig-1 mRNA levels was dependent on the dose of injected ASO with approximately 40% wig-1 mRNA reduction observed at 25 µg ASO and maximal (∼80%) reduction observed at 50 µg ASO. Furthermore, the duration of action of the wig-1 ASO lasted between 3 and 4 weeks following a single 75 µg dose (Figure 2B).

### Reduction in wig-1 levels promotes reduction in mutant HTT levels but fails to decrease p53 levels in vivo

**Figure 3:**
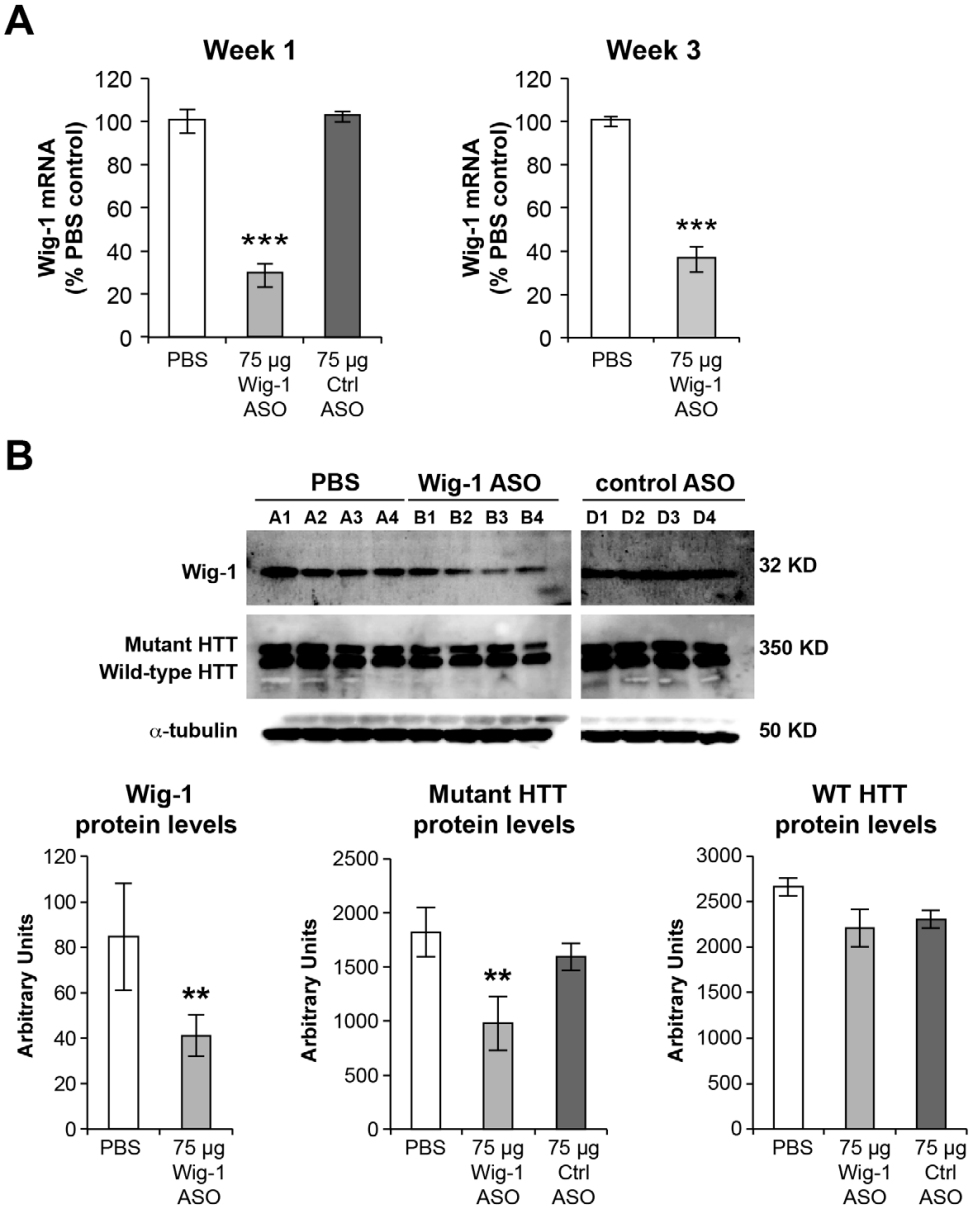
Effects of wig-1 ASO intrastriatal treatment on wig-1 and HTT levels in BACHD striatum. *5-month-old* male *BACHD* mice received 75 µg single bolus injection of wig-1 ASO, PBS, or control ASO in striatum. Animals were sacrificed after one, or three weeks. A) Striatum was harvested and total RNA was prepared from these sections, and used for real-time quantitative RT-PCR analysis to evaluate the expression of wig-1 mRNA. B) Tissue homogenates were prepared from striatum and used for analysis of Wig-1 and HTT protein levels. Data are expressed as means ± SEM (*n* = 4; **p≤0.05; ***p≤0.01). Letters A1–A4, B1–B4, D1–D4 refer to individual animals.

To determine the effects of wig-1 knockdown on htt expression, wig-1 ASO was administered as a single striatal injection (75 µg) in 5 month old BACHD mice, a model of Huntington's disease, and compared with PBS or a control oligonucleotide. BACHD mice express full length human mutant htt with 97 glutamine repeats under the control of endogenous htt regulatory elements. These mice exhibit progressive motor deficits, neuronal synaptic dysfunction, and late-onset selective neuropathology, which include significant cortical and striatal atrophy and neuronal degeneration. Animals were euthanized one or three weeks following the ASO administration, and wig-1 mRNA and protein levels were determined. Immunohistochemistry was used to confirm ASO distribution and to assess morphological changes in brain sections. Wig-1 mRNA levels were significantly reduced (∼70%) at both one and three weeks following ASO treatment whereas treatment with control oligonucleotide had no effect on wig-1 mRNA levels (Figure 3A). Furthermore, reduction in wig-1 protein levels was also observed in wig-1 ASO-treated samples with reductions comparable to the observed reductions in mRNA levels (Figure 3B). Interestingly, treatment with wig-1 ASO resulted in small (∼40%) but significant lowering of mutant htt protein levels (Figure 3B). However, levels of wild-type HTT protein were not significantly changed following wig-1 ASO treatment (Figure 3B).

**Figure 4:**
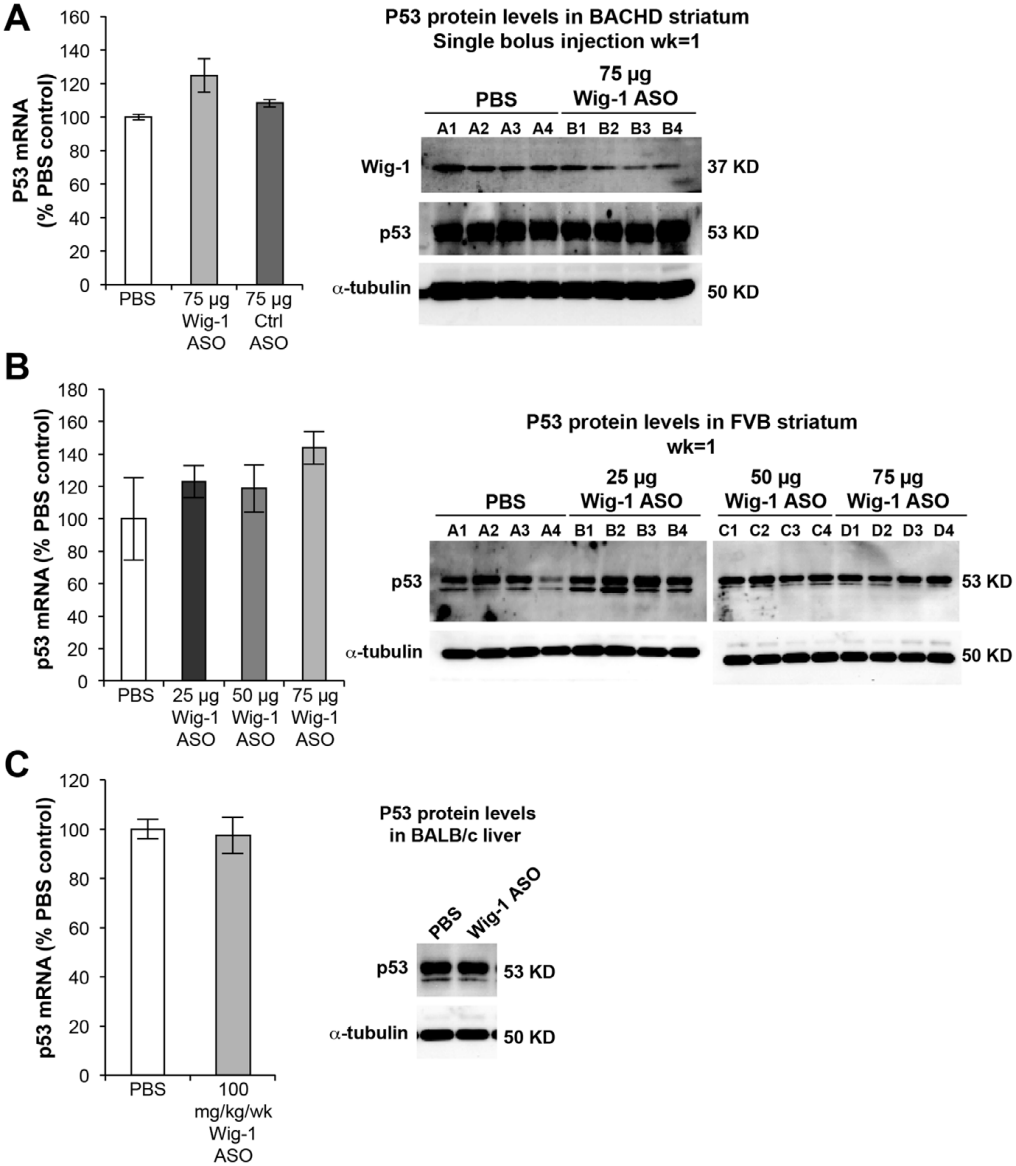
wig-1 knockdown does not affect p53 mRNA or protein levels following wig-1 ASO treatment in mice. *A) mRNA and protein were extracted from BACHD* striatum of mice treated with 75 µg of wig-1 ASO, PBS, or control ASO. B) p53 mRNA and protein levels in mouse FVB striatum following ASO injection with increasing doses of wig-1 ASO, and C) Effects of wig-1 ASO treatment on p53 mRNA and protein levels in mouse liver. Data are expressed as means ± SEM (*n* = 4).

The effects of wig-1 ASO treatment on p53 mRNA and protein levels was examined next in BACHD and FVB brain, and in BALB/c liver. Surprisingly, no reduction in p53 levels was observed in wig-1 ASO treated animals despite marked reduction in wig-1 levels. In fact, there was a slight trend for increased levels of p53 mRNA in BACHD and FVB striatum and p53 protein levels in FVB striatum in animals treated with wig-1 ASO (Figure 4A & B). Furthermore, whole cell lysate of BALB/c liver samples did not show any increase or decrease in p53 levels following wig-1 ASO treatment (Figure 4C). These findings suggest that wig-1 does not influence p53 mRNA or protein levels directly in mouse striatum and liver.

### Expression profile analysis of brain samples treated with wig-1 ASO

Gene array expression analysis was performed on BACHD mice treated with wig-1 ASO versus vehicle treated animals in order to gain insight into putative pathways affected by wig-1 suppression. Gene expression profiles of BACHD striatum were obtained in mice through whole genome microarray analysis. Genes with significant changes in expression following wig-1 ASO treatment were identified to determine potential gene pathways under control of wig-1 using, Ingenuity ™ Pathway Analysis software (Ingenuity Systems, CA, USA).

**Figure 5:**
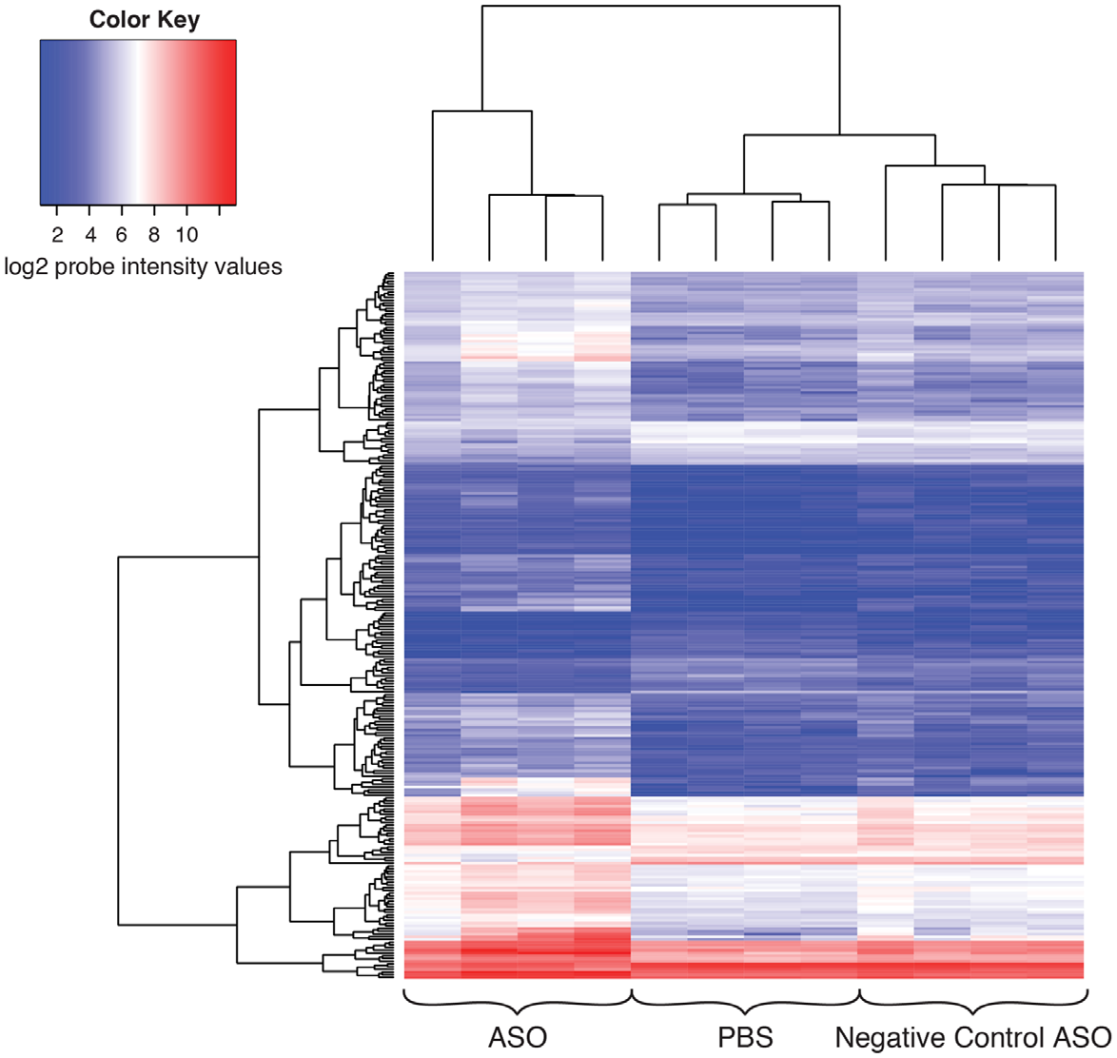
Heatmap of the *log2* normalized intensities of the significantly differentially expressed 260 gene probes on the array. The columns represent the PBS, ASO treated, and negative control samples and the rows represent the 260 differentially expressed gene probes on the array. The heatmap shows that the majority of the differentially expressed genes show mostly upregulation after ASO treatment. This is shown by the transition of probe intensities to higher values relative to the PBS and negative control samples for the 260 gene probes. The dendrogram on the left of the heatmap shows the clustering of genes having similar profiles across samples. The dendrogram on top of the heatmap shows the clustering of the samples. The PBS and negative control samples clustered together whereas the ASO treated samples clustered separately.

To improve the probability of successfully identifying novel pathways regulated by wig-1, a large number of genes displaying altered expression following wig-1 ASO treatment was obtained to support gene network analysis. We filtered on statistically significant genes (FDR cutoff 0.1) having −log_10_(p-values)> = 3.0 and absolute fold-changes > = 1.5. This filtering approach created a probe list of 260 genes that exhibited altered expression upon wig-1 ASO treatment. A hierarchical cluster analysis [18] of the log2 intensity profiles of the 260 genes is shown as a heatmap in Figure 5. This heatmap shows that both up-regulation and down-regulation of genes occurs in BACHD mouse striatum when treated with wig-1 ASO, with a larger portion of genes showing up-regulation. In fact, using the selection criteria described, 204 genes were found to be up regulated and 56 genes down regulated following wig-1 ASO treatment. Many genes which were significantly down-regulated are predicted to play a role in nervous system development and function, psychological disorders and cell-cycle control (table 1, and Figure S2). Furthermore, analysis of the up-regulated genes identified genes that are predicted to play a role in neurological disease and cell-to-cell signaling (table 2, and Figure S3).

**Table 1:** Genes down-regulated in BACHD mouse brain following wig-1 ASO treatment.

Down Regulated Genes	Accession number	Potential functions
zinc finger matrin type 3 (ZMAT3, wig-1)	NM_009517.2	Cell cycle, DNA replication, Recombination, and repair, cell cycle
autism susceptibility candidate 2 (AUTS2)	NM_177047.3	Cell cycle, DNA replication, Recombination, and repair, cell cycle
hemoglobin beta chain complex (HBB)	XM_921422.1	Cell cycle, DNA replication, Recombination, and repair, cell cycle
eukaryotic translation initiation factor 2C, 3 (EIF2C3)	NM_153402.2	Cell cycle, DNA replication, Recombination, and repair, cell cycle
integrin, beta-like 1 (ITGBL1)	NM_145467.2	Cell cycle, DNA replication, Recombination, and repair, cell cycle
potassium large conductance calcium-activated channel, subfamily M, alpha member 1(KCNMA1)	NM_010610.2	Cell cycle, DNA replication, Recombination, and repair, cell cycle
cyclin-dependent kinase 7 (CDK7)	NM_009874.3	Cell cycle, DNA replication, Recombination, and repair, cell cycle
protein kinase C, epsilon (PKCe)	NM_011104.3	Psychological disorders, cell morphology, cellular function and maintenance
amyloid beta (A4) precursor-like protein 2 (APLP2)	NM_001102455.1	Psychological disorders, cell morphology, cellular function and maintenance
latrophilin 3 (LPHN3)	NM_198702.2	Psychological disorders, cell morphology, cellular function and maintenance
polymerase (RNA) III (DNA directed) polypeptide H (POLR3H)	NM_030229.4	Psychological disorders, cell morphology, cellular function and maintenance
IMP2 inner mitochondrial membrane peptidase-like (S. cerevisiae) (IMMP2L)	NM_053122.3	Psychological disorders, cell morphology, cellular function and maintenance
roundabout homolog 2 (Drosophila) (ROBO2)	NM_175549.4	Cellular movement, nervous system development and function, cell-to-cell signaling and interaction
pleckstrin homology domain containing, family A member 5 (PLEKHA5)	NM_144920.3	Cellular movement, nervous system development and function, cell-to-cell signaling and interaction
glutamate receptor, ionotropic, delta 2 (GRID2)	NM_008167.2	Cellular movement, nervous system development and function, cell-to-cell signaling and interaction
UDP-Gal:betaGlcNAc beta 1,3-galactosyltransferase, polypeptide 1 (B3GALT1)	NM_020283.3	Cellular movement, nervous system development and function, cell-to-cell signaling and interaction
potassium voltage-gated channel, shaker-related subfamily, member 2 (KCNA2)	NM_008417.4	Cellular movement, nervous system development and function, cell-to-cell signaling and interaction

Ingenuity™ Pathway Analysis was used to ascribe down-regulated genes with potential functions (additional genes can be found in Figure S2).

**Table 2:** Genes up-regulated in BACHD mouse brain following wig-1 ASO treatment.

Up Regulated Genes	Accession number	Potential functions
B-cell leukemia/lymphoma 2 related protein A1a (BCL2A1)	NM_009742.3	Genetic disorder, neurobiological disease, cell-to-cell signaling and interaction
PYD and CARD domain containing (PYCARD)	NM_023258.4	Genetic disorder, neurobiological disease, cell-to-cell signaling and interaction
sterol O-acyltransferase 1 (SOAT1)	NM_009230.3	Genetic disorder, neurobiological disease, cell-to-cell signaling and interaction
caspase 8 (CASP8)	NM_001080126.1	Cardiovascular disease, organismal injury and abnormalities, inflammatory response
interferon-induced protein with tetratricopeptide repeats 3 (IFIT3)	NM_010501.2	Cardiovascular disease, organismal injury and abnormalities, inflammatory response
apolipoprotein E (APOE)	NM_009696.3	Cardiovascular disease, organismal injury and abnormalities, inflammatory response
arachidonate 5-lipoxygenase activating protein (ALOX5AP)	NM_009663.1	Neurobiology disease, endocrine system disorders, metabolic disease
extracellular matrix protein 1(ECM1)	NM_007899.2	Neurobiology disease, endocrine system disorders, metabolic disease
intercellular adhesion molecule 1(ICAM1)	NM_010493.2	Neurobiology disease, endocrine system disorders, metabolic disease
glycoprotein (transmembrane) nmb (GPNMB, osteoactivin)	NM_053110.4	Cancer, antigen presentation, cellular movement
N-acetylneuraminic acid synthase (sialic acid synthase) (NANS)	NM_053179.3	Cancer, antigen presentation, cellular movement
asparaginase homolog (S. cerevisiae) (ASPG)	NM_001081169.1	Cancer, antigen presentation, cellular movement
ATP-binding cassette, sub-family C (CFTR/MRP), member 3 (ABCC3)	NM_029600.3	Cell cycle, cellular development, gene expression
carnitine palmitoyltransferase 2 (CPT2)	NM_009949.2	Cell cycle, cellular development, gene expression
transmembrane emp24 domain containing 3 (TMED3)	NM_025360.2	Cell cycle, cellular development, gene expression

Ingenuity™ Pathway Analysis was used to ascribe up-regulated genes with potential functions (additional genes can be found in Figure S3).

### PCR and western blot confirmation

**Figure 6:**
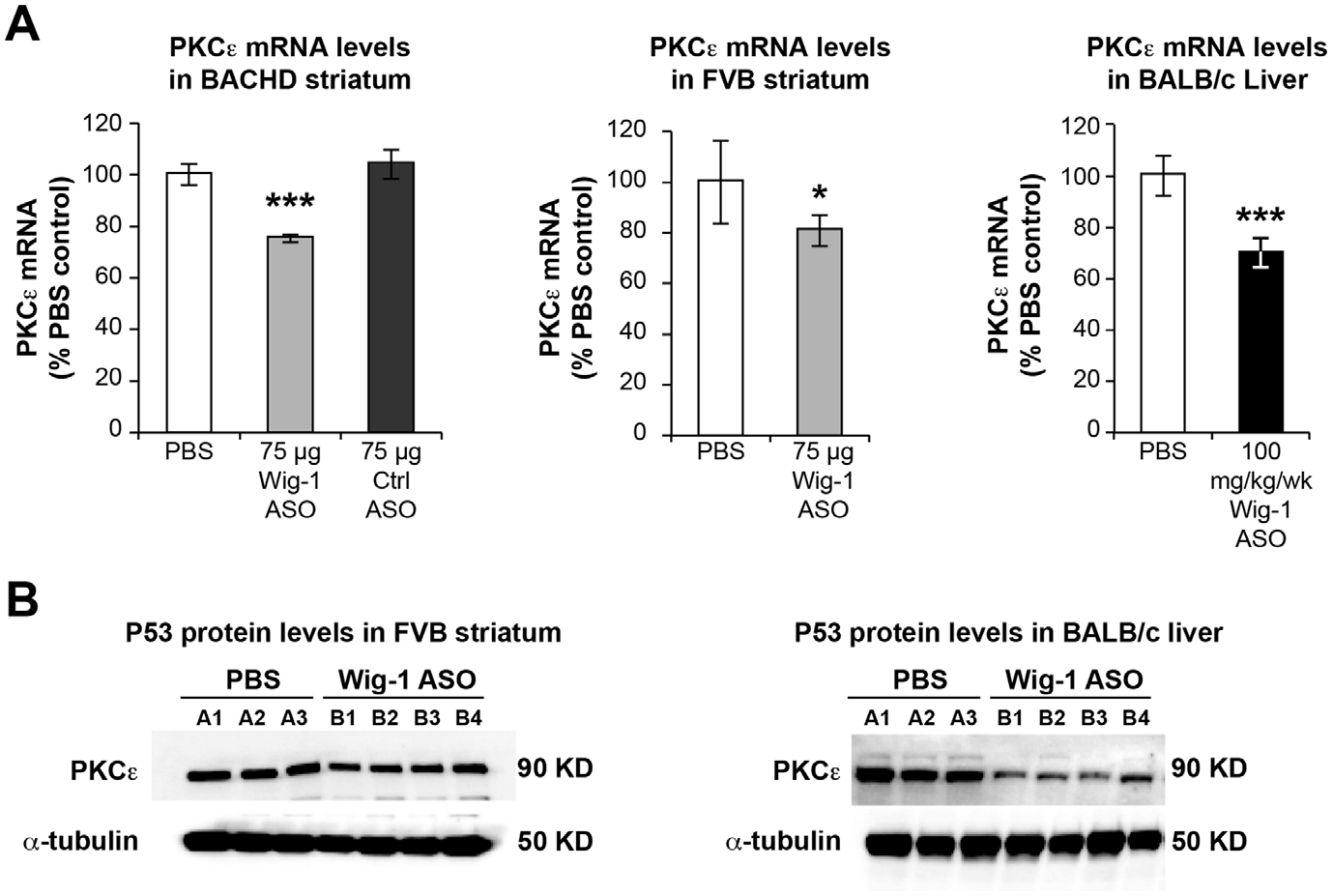
Effects of wig-1 ASO treatment on PKCε levels. PKCε mRNA and protein levels in BACHD and FVB striatum, and BALB/c liver were determined in animals treated with wig-1 ASO and compared to control animals. A) Effects of wig-1 ASO treatment on PKCε *mRNA levels.* *B*) Effects of wig-1 ASO treatment on PKCε *protein levels*. Data are expressed as means ± SEM (*n* = 4; **p≤0.05; ***p≤0.01).

**Figure 7:**
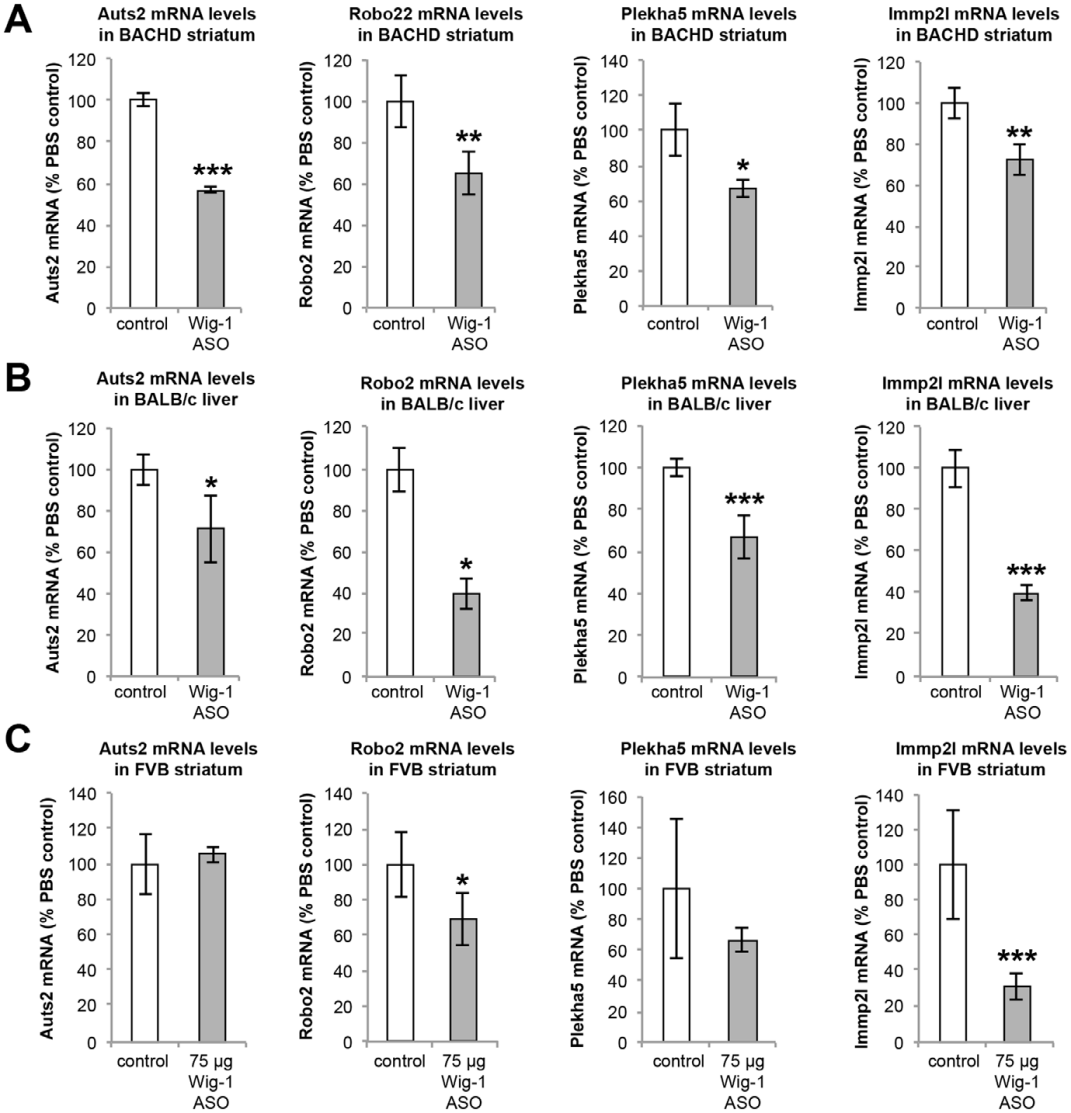
Real Time-PCR confirmation of down-regulated mRNAs following wig-1 ASO treatment in mice. mRNA levels of *Auts2, Robo2, Plekha5, and IMMP2L* in (A) BACHD striatum, (B) BALB/c liver, or (C) FVB striatum was compared to control animals. Data are expressed as means ± SEM (*n* = 4; *p≤0.1; **p≤0.05; ***p≤0.01).

RT-PCR confirmation of microarray results was performed on a subset of genes identified as being down regulated following wig-1 ASO treatment. Specifically, five genes (PKCε, AUTS2, ROBO2, PLEKHA5, and IMMPL2) were selected for confirmation. Consistent with microarray results, all of these genes showed down-regulation in BACHD and FVB brain as well as BALB/c liver in animals treated with wig-1 ASO, as compared to control samples. For example, PKCε which is highly expressed in both mouse brain and liver was significantly reduced at both the RNA and protein levels in BACHD and FVB brain, and in BALB/c liver (p≤0.1 and p≤0.01 respectively) (Figure 6). Wig-1 ASO treatment produced significant reductions in AUTS2 mRNA levels in BACHD brain (p≤0.01) (Figure 7). A trend in reduction of AUTS2 mRNA level was observed in BALB/c liver (p≤0.1) whereas no reduction in AUTS2 levels was observed in non-BACHD (FVB) mouse brain. Autism susceptibility candidate 2 (Auts2) is a gene associated with autism and mental retardation [19], [20] whose exact function is unknown, but is expressed in different regions of the mouse brain.

Roundabout axon guidance receptor homolog 2 (ROBO2) [21], [22], [23] is also expressed in brain and liver. Intrastriatal injection of wig-1 ASO in BACHD and FVB mice resulted in significant reductions of ROBO2 mRNA levels in both BACHD and FVB striatum (p≤0.05 and p≤0.1 respectively) (Figure 7). Furthermore, systemic administration of the wig-1 ASO also resulted in marked reduction of ROBO2 mRNA levels in BALB/c liver (p≤0.1).

Another gene that was found to be down regulated in BACHD brain and BALB/c liver (p≤0.1 and p≤0.05 respectedly) following treatment with wig-1 ASO was the Pleckstrin homology domain containing family A member 5 (PLEKHA5), which is expressed in different tissues, with the highest level of expression in prefrontal cortex, fetal brain, uterus, and adrenal gland (Figure 7). Furthermore, a statistically significant decrease in expression of the inner mitochondrial membrane peptidase 2-like (IMMP2L) [24], [25] gene was also observed in BACHD (p≤0.05) and FVB (p≤0.01) striatum following wig-1 ASO treatment, which has been linked to a possible rare cause of Autism and Gilles de la Tourette syndrome (GTS) [25]. Reductions in IMMP2L mRNA levels were also observed in BALB/c liver following systemic treatment with wig-1 ASO (p≤0.01) (Figure 7).

**Figure 8:**
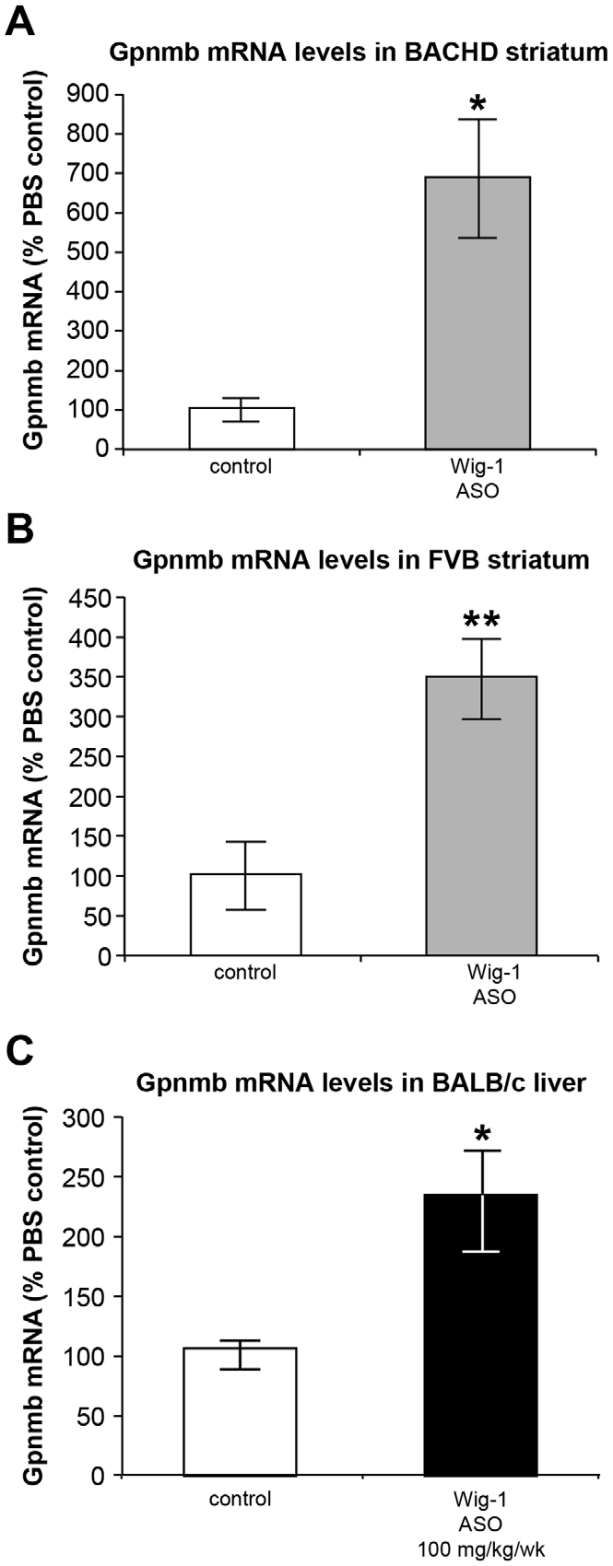
Up-regulation of Gpnmb mRNA levels following wig-1 ASO treatment. A) Gpnmb mRNA levels in BACHD striatum; B) FVB striatum, and C) BALB/c liver. Data are expressed as means ± SEM (*n* = 4; *p≤0.1; **p≤0.05).

Glycoprotein transmembrane nmb (gpnmb) is expressed widely in tissues including brain, liver, retina, skin, placenta, and salivary gland. Following striatal wig-1 ASO treatment, a marked elevation of gpnmb mRNA levels was observed in BACHD (p≤0.1) and FVB (p≤0.05) striatum (Figure 8). Furthermore, elevations in gpnmb mRNA levels were also observed in BALB/c liver following systemic treatment with wig-1 ASO (p≤0.1) (Figure 8).

## Discussion

wig-1 is a regulatory protein that has been shown to function as a transcription factor, an RNA binding protein, and a regulator of both RNA and protein stabilization [1], [4], [9]. Furthermore, wig-1 has also been shown to have a complex relationship with the tumor suppressor p53, with reports indicating that p53 can control levels of wig-1 and that wig-1 can regulate levels of p53, suggesting that p53 and wig-1 tightly control each other's expression in certain cell systems [9]. Moreover, p53 has been linked with expression levels of the Huntington gene and with the Huntington phenotype in mice [11], [26]. Therefore, we investigated the effects of wig-1 on p53 levels, on htt expression, and on gene expression more broadly using microarray analysis in mouse brain and liver using highly specific wig-1 antisense oligonucleotides (ASOs). ASOs were administered to normal BALB/c mice systemically and in BACHD and FVB mice by intrastriatal injection, and effects on gene expression were examined. Since ASOs do not cross the blood brain barrier [27], [28] local administration is required for ASO-mediated activity in the CNS. Direct ASO administration to the striatum resulted in broad distribution with strong uptake in both neuronal and glial cells, with the intensity of ASO uptake being greatest in the proximity of the ASO injection site. Moreover, marked reductions in wig-1 mRNA and protein levels was demonstrated in the BACHD and FVB striatum and in BALB/c liver, and this reduction in wig-1 levels was well tolerated over a four week period.

wig-1 has been reported to bind to p53 mRNA *in vitro*, causing stabilization of the p53 message [9]. Accordingly, siRNA-mediated reduction in wig-1 levels resulted in a corresponding reduction in p53 levels in cell culture [9]. However, in studies reported here, suppression of wig-1 levels in mouse striatum or in mouse liver had no significant effect on p53 mRNA or protein levels. The reason behind this discrepancy is unclear, but most likely is related to differences between the cell culture system and animals, or the specific cell types examined. Certainly, cell culture may not be an accurate representation of gene regulation in animals. Additionally, the effects of wig-1 on p53 mRNA levels may differ between different cell types, such as striatal cells (i.e., neuronal and glial cells) and hepatocytes as described here relative to the osteosarcoma cells and fibroblasts that were investigated in the prior report. Interestingly, ASO-mediated suppression of endogenous wig-1 levels in the striatum of BACHD mice led to a significant reduction (approximately 50%) in mutant HTT protein levels with no significant effect on the levels of endogenous wild type HTT levels. The mechanisms underlying this result are not yet clear. However, considering the fact that we have not seen significant changes in mutant HTT mRNA levels despite reductions in mutant protein levels suggests that wig-1 may be regulating mutant HTT levels post-transcriptionally (data not shown). Nevertheless, more studies are needed to establish the link between wig-1 and mutant HTT protein expression.

Despite the lack of effects on p53 mRNA and protein levels in mouse liver and striatum following wig-1 suppression, broad changes in mRNA levels was observed following wig-1 suppression in mouse striatum based on microarray analysis. Our results suggest that wig-1 can regulate gene expression through both gene repression and activation, as reflected by both down-regulation and up-regulation of genes following wig-1 ASO suppression. We identified 204 genes that were up-regulated and 56 genes that were down-regulated following wig-1 ASO treatment. These genes have been linked to a broad range of putative cellular functions including cell cycle regulation, DNA replication, cell survival, and neurological function.

Wig-1 suppression promoted confirmed changes in two genes linked to cancer. Wig-1 ASO treatment resulted in decreases in PKCε levels in both mouse brain and liver. PKCε signaling is involved in cell invasion, motility, proliferation, and survival, and has been linked to malignancies of the central nervous system [29]. The role of PKCε in cancer promotion is believed in part to involve the ras signaling pathway [30], [31], [32] and the regulation of expression of specific Bcl-2 family members [33]. Wig-1 suppression also caused a significant upregulation in expression of gpnmb in mouse brain and liver, a transmembrane glycoprotein also known as osteoactivin or HGFIN. Gpnmb has been implicated as a tumor suppressor, and has been reported to promote cell transformation and proliferation, and loss of cell contact dependency [34], [35], [36]. Furthermore, high expression of gpnmb has been associated with aggressive melanoma, glioma and breast cancer [37], [38], [39], [40]. Interestingly, wig-1 has also been reported to regulate tumor cell apoptosis and cell proliferation [2], [3]. Our findings suggest potential genes and mechanisms relevant to cancer pathways that wig-1 may be acting through.

Our results also suggest a role for wig-1 in regulating gene expression within specific pathways relevant to CNS function and disease. Wig-1 suppression led to a reduction in expression of numerous genes in mouse brain and liver. Genes down-regulated and confirmed by RT-PCR included AUTS2, ROBO2, and IMMP2L. AUTS2 (Autism susceptibility candidate 2) is a nuclear protein that is highly expressed in developing neurons of certain brain regions, notably the frontal cortex and cerebellum, and has been linked with the neuropathy of autism [19], [41]. ROBO2 (roundabout axon guidance receptor homolog 2) is a receptor for SLIT1 ligand, which is critically important for axon guidance and in CNS development [42], [43], [44]. IMMP2L (inner mitochondrial membrane peptidase 2-like) is a mitochondrial peptidase believed to be involved in polypeptide precursor processing within the inner mitochondrial membrane and has been shown to be critical for normal mitochondrial function through knockout mouse studies (Lu et al.). Interestingly, the IMMP2L locus has been linked with Autism Spectrum Disorders (ASDs) [24] and with Tourette Syndrome [45], [46], [47]. Regulation of expression of both AUTS2 and IMMP2L by wig-1 suggests a possible role for wig-1 in autism.

The mechanism by which wig-1 regulates the expression of genes shown to be modulated following wig-1 ASO treatment is unknown. However, wig-1 has been shown to bind to the promoter of some genes through its C2H2 zinc finger motifs and thereby regulate transcription [1]. More recently, regulation of RNA stability through binding of wig-1 to putative RNA sequence motifs within the 3′UTR of certain RNAs, including p53 [9], have been proposed based on similarities between the wig-1 zinc finger RNA binding motif and those of a small group of dsRNA-binding proteins [5]. Interestingly, examination of the 3′UTR of wig-1 regulated mRNAs identified in this study revealed the presence of sequence motifs (e.g., UUAUUUAUU and AUUUAAUUUA) that have been linked with RNA: protein binding and regulation [48], [49], [50], [51].

In summary, our studies indicate that wig-1 regulates gene expression broadly in mouse brain and liver through mechanisms that appear independent of p53. Our findings indicate that reduction of wig-1 by ≥80% in adult mouse brain and liver are generally well tolerated, and suggest that wig-1 can positively and negatively regulate the expression of genes implicated in various cellular pathways and pathologies including cancer and neurodegenerative diseases. Furthermore, a novel role for wig-1 in the posttranscriptional regulation of mutant huntingtin protein levels may have been identified.

## Materials and Methods

### 2^nd^ generation Antisense Oligonucleotides (ASOs) chemistry

All oligonucleotides were 20 nucleotides in length and chemically modified with phosphorothioate in the backbone and 2′-*O*-methoxyethyl (MOE) on the wings with a central deoxy gap (“5-10-5” design). Oligonucleotides were synthesized using an Applied Biosystems 380B automated DNA synthesizer (Perkin Elmer-Applied Biosystems, Foster City, CA, USA) and purified. A negative control oligonucleotide, which has the same chemical composition as the wig-1 ASO but no complementarities to any known gene sequence, was also included in the studies.

### Identification and characterization of ISIS wig-1 ASOs in vitro and in vivo

To identify mouse wig-1 antisense inhibitors, rapid-throughput screens were performed in the bEND cell line (ATCC, CRL-2299™). In brief, 80 ASOs were designed to the wig-1 mRNA sequence, all of which targeted binding sites within the coding region of the wig-1 mRNA. The reduction of target gene expression was analyzed with real-time quantitative RT-PCR after transfection of the cells with ASOs for 24 h. Based on target reduction, 4 ASOs were selected and further characterized in a dose-response screen. The most potent ASOs from the screen were chosen, and their in vivo activity was confirmed by intraperitoneal (IP) administration in BALB/c mice. The most potent ASO was chosen as the wig-1 ASO for subsequent studies.

### Animal studies

This study was conducted in accordance with the guidelines of the Institutional Animal Care and Use Committee at Isis Pharmaceuticals and approved by the committee. Protocol number and PHS Assurance numbers are P-0190 and A4318-01, respectively. Six-week-old male BALB/c mice were injected with four wig-1 ASOs at 100 mg/kg/week or saline twice/week for 4 weeks. ASOs were dissolved in PBS and administered intraperitoneally. Animals were euthanized 48 hours after the last dose. Blood, liver, kidney and spleen were collected to measure toxicity, PK, as well as RNA and protein levels.

### Treatment and surgery

Groups of four FVB (8 weeks of age) and BACHD mice (5-months-old) were treated with wig-1 ASO at a dose of 25, 50, or 75 µg delivered by single striatal bolus injection. Control groups of four FVB and BACHD mice were similarly treated with PBS or with negative control oligonucleotide. Mice were individually anaesthetized with 3% isoflurane and were maintained throughout the surgical procedure in an ASI small animal stereotaxic system (ASI Instruments, SAS-4100) with a gas nose cone delivering 2% isoflurane. The scalp of the animal was sterilized with iodine solution followed by 70% ethanol. Then, a longitudinal mid-saggital incision 1 cm in length was made in the scalp. Hamilton gas tight 10 µL Syringe with removable 26 gauge needle by advancing the end of the needle through the scull to the appropriate coordinate. Coordinates used: 0.5 mm anterior, 2.0 mm lateral on right and 3.0 mm deep from Bregma with flat skull nosebar setting. Different concentrations of ASO in a total volume of two µl/concentration were administered by injection. After one, two, three and four weeks the mice were euthanized using isoflurane followed by decapitation. Brain tissue, including the striatum, was extracted for protein and RNA analysis. Histopathology was conducted to assure the distribution of ASO as well as safety.

### Real-time quantitative PCR

RNA was extracted by using a QIAGEN RNAeasy kit (QIAGEN). The mRNA was transcribed to cDNA by using MuLV reverse transcriptase (New England Bio Labs). The abundance of transcripts was assessed by real-time PCR on a 7700 Fast Real-Time PCR System (Applied Biosystems). Each run was evaluated in triplicate for both the gene of interest and endogenous control for mRNA levels, Cyclophilin A (Figure S1). The expression data for the gene of interest were normalized for the efficiency of amplification determined by the standard curve included for each data acquisition.

### Immunoblotting

For Western blots, samples were lysed in 500 µL of extraction buffer [50 mM Tris (pH 8), 150 mM NaCl,1% NP-40, 0.5% Sodium deoxycholate, 1 mM sodium orthovanadate, 1 mM NaF, protease inhibitor cocktail set III, EDTA-free (539134; EMD CHEMICALS, INC.)]. The homogenate was centrifuged at 14,000× *g* for 10 min at 4°C to pellet insoluble material. The supernatant was saved as the final lysate and stored at −80°C. Fifty micrograms of lysate were electrophoresed through 4–20% (for wig-1 protein) or 12% (for p53 protein) Tris-Glycine gel and transferred to nitrocellulose membrane (iBlot, Invitrogen). The membrane was blocked in 5% milk for one hour and then incubated with either α-rabbit ZMAT3 (ARP50793_P050, Aiva Systems Biology), α-mouse p53, 1C12 (2524, Cell Signaling), α-rabbit PKCε, 22B10 (2683, Cell Signaling), anti-mouse huntingtin (MAB2166, Millipore) or α-mouse α-tubulin, DM1A (ab7291, Abcam) overnight. The membrane was washed extensively and then incubated with either anti-rabbit IgG or anti-mouse IgG (GE healthcare UK limited). Chemiluminescence detection reagents (ECL Plus; Amersham Pharmacia) enabled visualization of peroxidase reaction products, and quantified using the software ImageJ.

### Immunohistochemistry

For hemotoxylin and eosin (H & E) staining, pieces of liver from *BALB/c* mice were fixed in 10% buffered formalin and embedded in paraffin wax. Brain sections were also fixed in 10% formalin. Multiple adjacent 4-µm sections were cut and mounted on glass slides. After dehydration, the sections were stained. Images of the histological sections were analyzed.

### Microarray gene expression analysis

Total RNA was extracted from BACHD striatum, treated with wig-1 ASO, negative control ASO, or PBS, using RNeasy Plus mini kits (Qiagen). Gene expression was analyzed by hybridization to the MouseWG-6 v2 Expression BeadChip array (Illumina), and array data analysis was performed using Bioconductor packages *lumi* and *siggenes* [52]. The entire microarray data set has been submitted to GEO [53] and can be referenced via accession ID GSE29751. Siggenes was used to determine probes showing statistically significant differential expression at an FDR (False Discovery Rate) cutoff of 0.100. Gene network prediction was determined by using Ingenuity™ Pathway Analysis (IPA) (Ingenuity Systems, CA, USA).

## Supporting Information

Figure S1 **List of primers used for real-time PCR.**
(DOCX)

Figure S2 **Gene network pathways identified by Inegenuity Pathway Analysis for down-regulated genes.** The table above identifies the genes within a network and includes a score, which is used to rank the networks. The genes within each network are comprised of genes identified by microarray analysis in addition to other genes within the network as identified by Ingenuity Pathway Analysis. The total number of genes identified as down-regulated via microarray analysis within each network is identified in the column labeled “Focus Molecules”. The score for each network is obtained from the *−log_10_(p-value)*, where the p-value is obtained from a Fisher Exact Test. The score ranks the networks based on the probability of obtaining the same networks by chance when sampling a similar number of genes from the Ingenuity Knowledge Base. Network scores with a high value (> = 2) are more significant.
(DOCX)

Figure S3 **Gene network pathways identified by Inegenuity Pathway Analysis for up-regulated genes.** The table above identifies the genes within a network and includes a score, which is used to rank the networks. The genes within each network are comprised of genes identified by microarray analysis in addition to other genes within the network as identified by Ingenuity Pathway Analysis. The total number of genes identified as up-regulated via microarray analysis within each network is identified in the column labeled “Focus Molecules”. The score for each network is obtained from the *−log_10_(p-value)*, where the p-value is obtained from a Fisher Exact Test. The score ranks the networks based on the probability of obtaining the same networks by chance when sampling a similar number of genes from the Ingenuity Knowledge Base. Network scores with a high value (> = 2) are more significant.
(DOCX)
