# Supplementary material for: Genomic Analysis of wig-1 Pathways
Source: PLoS One. 2012 Feb 7;7(2):e29429. doi: 10.1371/journal.pone.0029429 (PMC3274543; doi:10.1371/journal.pone.0029429)
Supplement: Figure S1 — List of primers used for real-time PCR. (DOCX) [file pone.0029429.s001.docx]

**Supplementary Figure S1. List of primers used for real-time PCR**

| **set name** | **sequence** |
| --- | --- |
| wig-1 For | TGGCCACCAGGAGGAATATG |
| wig-1 Rev | GGGAGCGGGCATTGAAGT |
| wig-1 probe | ATCCAGTCCAGAGCAATTCAGGCCCTX |
| p53 For | GCTTCTCCGAAGACTGGATGA |
| p53 Rev | AAGGAGTTTCCATAAGCCTGAAAA |
| p53 probe | TCACAGTCGGATATCAGCCTCGAGCTCX |
| PKCε For | GCCAACTGCACCATCCAGTT |
| PKCε Rev | CAGGTCAATCCAGTCCTCGAA |
| PKCε probe | AGCTGCTGCAGAATGGGAGCCGTX |
| IMMP2L For | TGATCGCTCTTGAAGGAGATATTG |
| IMMP2L Rev | TCCATGATGATCGCCTTCAAC |
| IMMP2L probe | AACCGGTTGGTCAAAGTCCCCCGX |
| PLEKHA5 For | CCAGAGAGGAGATGTGACGATAGA |
| PLEKHA5 Rev | CCAGCTGGCATTGACCTTCT |
| PLEKHA5 probe | CGCAGGCACAGGCCTCATCACCX |
| AUTS2 For | TGACCCATTCTACCGGCATAG |
| AUTS2 Rev | CCCGTGGGTGGGATCAT |
| AUTS2 probe | CTCTTCCACTCCTATCCTCCTGCGGTCTX |
| ROBO2 For | CAAGAGATCAGATCGTTGCTCAA |
| ROBO2 Rev | CTGCCTTCTTTCTGCCAAAAA |
| ROBO2 probe | CCGAACAGTGACATTCCCCTGTGAAACX |

For: Forward; Rev: Reverse
