# Supplementary material for: Genomic Analysis of wig-1 Pathways
Source: PLoS One. 2012 Feb 7;7(2):e29429. doi: 10.1371/journal.pone.0029429 (PMC3274543; doi:10.1371/journal.pone.0029429)
Supplement: Figure S2 — Gene network pathways identified by Inegenuity Pathway Analysis for down-regulated genes. The table above identifies the genes within a network and includes a score, which is used to rank the networks. The genes within each network are comprised of genes identified by microarray analysis in addition to other genes within the network as identified by Ingenuity Pathway Analysis. The total number of genes identified as down-regulated via microarray analysis within each network is identified in the column labeled “Focus Molecules”. The score for each network is obtained from the −log10(p-value), where the p-value is obtained from a Fisher Exact Test. The score ranks the networks based on the probability of obtaining the same networks by chance when sampling a similar number of genes from the Ingenuity Knowledge Base. Network scores with a high value (> = 2) are more significant. (DOCX) [file pone.0029429.s002.docx]

**Supplementary Figure S2**

| **Molecules in Network** | **Score** | **Focus Molecules** | **Top Functions** |
| --- | --- | --- | --- |
| A830091E24, AHR, ATG10, AUTS2 (includes EG:26053), beta-estradiol, CAK, CD163, CDC42EP4, CDK7, CTSB, DICER1, EIF2C3, ERCC2, ERCC5, HBB (includes EG:3043), HINT1, HNRNPA2B1, IFNG, ITGBL1, KCNMA1, KCNMB1, MED21, MIRLET7C, MNAT1, NETO2, NME1, RECQL4, SPHK1, TFIIH, TP53, USP7, USP29, XPC, ZMAT3, ZRANB2 | 26 | 12 | Cell Cycle, DNA Replication, Recombination, and Repair, Cell Death |
| ADAP1, ANKRA2, APLP2, C15ORF29, CACNA1B, CARS, CASK, CNTNAP2, CREB1, Gpcr, GRIK1, GRIK3, GRIP1, GRM2, GRM3, GRM4, GRM6, GTF2H3, HNF4A, HSPA5, KIF17, LPHN3, Ncx, PAD4, PDLIM5, Pkc(s), POLR3E, POLR3G, POLR3H, PRKCE, PRLHR, RGS6, SRSF11, tridecanoic acid, UGT1A7 | 21 | 10 | Psychological Disorders, Cell Morphology, Cellular Function and Maintenance |
| A2BP1, APBA2, ARF3, ATN1, B3GALT1, C13ORF23, CPE, DIAPH2, DIO2, DLG4, GAS7, glycine, GRIA3, GRID2, GRIK1, GRIK2, GRIK5, KCNA2, KCNAB2, KIF1B, LYVE1, MMP2, PCDHA13, PLEKHA5, PRDX2, retinoic acid, ROBO1, ROBO2, SLC36A2, SLIT1, SLIT2, SPOCK3, TNF, Vegf, WSB1 | 21 | 10 | Cellular Movement, Nervous System Development and Function, Cell-To-Cell Signaling and Interaction |
| LMTK3, ZBTB16 | 3 | 1 | Embryonic Development, Organismal Development, Skeletal and Muscular System Development and Function |
| NKAIN2, REST | 3 | 1 | Cell Death, Gene Expression, Cardiac Arrythmia |
| asparaginase, GTF2A1, MIR330 (includes EG:100313974), MLL, TASP1 | 2 | 1 | Cellular Development, Cellular Growth and proliferation |
