# Supplementary material for: Genomic Analysis of wig-1 Pathways
Source: PLoS One. 2012 Feb 7;7(2):e29429. doi: 10.1371/journal.pone.0029429 (PMC3274543; doi:10.1371/journal.pone.0029429)
Supplement: Figure S3 — Gene network pathways identified by Inegenuity Pathway Analysis for up-regulated genes. The table above identifies the genes within a network and includes a score, which is used to rank the networks. The genes within each network are comprised of genes identified by microarray analysis in addition to other genes within the network as identified by Ingenuity Pathway Analysis. The total number of genes identified as up-regulated via microarray analysis within each network is identified in the column labeled “Focus Molecules”. The score for each network is obtained from the −log10(p-value), where the p-value is obtained from a Fisher Exact Test. The score ranks the networks based on the probability of obtaining the same networks by chance when sampling a similar number of genes from the Ingenuity Knowledge Base. Network scores with a high value (> = 2) are more significant. (DOCX) [file pone.0029429.s003.docx]

**Supplementary Figure S3**

| **Molecules in Network** | **Score** | **Focus Molecules** | **Top Functions** |
| --- | --- | --- | --- |
| BCL2A1, BCR, C1q, C1QA, C1QB, C1QC, Complement component 1, DEF6, ENPP1, FAM3B, Fc gamma receptor, Fc receptor, FCGR2A, FCGR2B, FCGR3A, IgG, IgG1, IgG2a, Igm, LAPTM5, LCN2, LGALS1, MHC Class I (complex), MHC CLASS I (family), NADPH oxidase, NFkB (complex), NFkB (family), PYCARD, SAA, SOAT1, SYK/ZAP, TLR2/3/4/9, TREM2, TRIP6, TYROBP | 32 | 18 | Genetic Disorder, Neurological Disease, Cell-To-Cell Signaling and Interaction |
| Akt, Ap1, APOE, B2M, CASP8, collagen, CTSS, CTSZ (includes EG:1522), GCNT1, GSTM1, H-2db, HLA Class I, HLA-C, HLA-DQB1, HMOX1, IFIT3, IFITM1, IFITM2, IFITM3, IFN Beta, Immunoglobulin, Interferon alpha, IRF9, IRG, MHC, MHC Class II (complex), NFAT (complex), Nfat (family), NfkB1-RelA, P38 MAPK, PARVG, TCR, TNFRSF1A, UNC93B1, WAS | 30 | 19 | Cardiovascular Disease, Organismal Injury and Abnormalities, Inflammatory Response |
| Actin, ALOX5AP, Ap2 alpha, BGLAP, Calpain, Caspase, Cyclin A, ECM1, FSH, GFAP, hCG, HIST1H3A, HIST4H4, Histone h3, Histone h4, HK2, ICAM1, Iga, Insulin, Jnk, Lh, LIN37, LY9, PLIN2, RNA polymerase II, Rxr, S100A6, S100A11, Sod, TCN2, Tgf beta, TGFB1, TSPO, UPP1, Vegf | 29 | 17 | Neurological Disease, Endocrine System Disorders, Metabolic Disease |
| AHNAK, ARHGDIB, CCL2, CCL4, CCL8, CCL21, CD22, CSF3R, CYBA, FES, GBP2, Gm-csf, Growth hormone, Ifn, Ifn gamma, IL1, IL12 (complex), LDL, LRP, NCF1, P110, p85 (pik3r), Pdgf, PDGF BB, Pdgfr, PI3K (complex), PLC gamma, Sapk, SLC7A7, SOCS3, STAT5a/b, TGM2, TIMP1, Tnf, Tnf receptor | 27 | 16 | Cellular Movement, Hematological System Development and Function, Immune Cell Trafficking |
| 3-beta,17-beta-androstanediol, ANXA11, ASPG, Ca2+, CCL22, CD38, Cyclooxygenase, ESM1, ESR1, FCGR3A, GBP4 (includes EG:115361), GPNMB, GREB1, GUCA, GUCA1A, GUSB, HEXB, HIST1H2BH, HSP90AB1, ICOSLG, IFNG, KCNIP3, KCNK6, KDM5A, LAMP1, LYZ, M6PR, MAN2B1, Mannosidase Alpha, MED7 (includes EG:9443), NANS, POU2F2, RAET1B, SLC15A3, TBP | 27 | 16 | Cancer, Antigen Presentation, Cellular Movement |
| ARPC1B, CD63, CD68, CELA1, Collagen type I, Collagen type IV, Collagen(s), DCT, Elastase, ERK1/2, Fcer1, FCGR1A/2A/3A, Fibrin, Fibrinogen, GRN, GUSB, HEXA, HEXB, Ifnar, Ige, Integrin, Integrin alpha V beta 3, ITGB5, Kallikrein, KLK6, Laminin, Laminin1, LGALS3, LGALS3BP, MED7 (includes EG:9443), Mek, PLAU, SERPING1, SPP1, VitaminD3-VDR-RXR | 27 | 17 | Cell-To-Cell Signaling and Interaction, Carbohydrate Metabolism, Small Molecule Biochemistry |
| ABHD5, ABI3, AIF1, ANXA3, APOC4, ARAF, ARNT2, ARPC1B, beta-estradiol, CNN2, COPZ2 (includes EG:51226), CYTH4, FEZF1, FGF6, GNB1, GNGT2, HRAS, ICMT, IPCEF1, KIAA1274, KRAS, LCN2, MAPK1, MIR124, MPEG1, PAK1, phosphatidylinositol-3,4,5-triphosphate, RLN2, RSU1, SERPINB6, SGPL1, SHOC2, SPRY1, SYNGR2, ZDHHC18 | 25 | 15 | Cell Signaling, Connective Tissue Development and Function, Skeletal and Muscular System Development and Function |
| ABCE1, ACY3, BAZ1A, BAZ1B, C2, C1S, C4A, CTSA, CTTNBP2, CTTNBP2NL, CXADR, ERCC5, EWSR1, FAM40A, FAM40B, HNF4A, KLHL6, MBL2, MFSD1, MYL6, MYO1A, MYO1F, PDCD10, POLD4, RBM15, RFC5, RNASE2, RNASE4, SEPX1, SIKE1, STK24, SUPT16H, TCIRG1, TMEM176A, USP46 | 22 | 14 | Antigen Presentation, Humoral Immune Response, Inflammatory Response |
| ABCC3, ALDH3B1, BAMBI, CAR ligand-CAR-Retinoic acid-RXRα, CD38, CDKN2A, COTL1, CPT2, CPT1B, EMP3, ENTPD5, FANCL, FCN2, GFRA1, GPRC5A, HAS1, KIF23, KIF3C, LGALS9, LOXL2, Ncoa-Nr1i2-Rxra, Ncoa-Nr1i3-Rxra, retinoic acid, RXRA, S100A11, SMARCA4, STARD10, TIA1, TIAL1, TMED3, TNF, TNNI2, TREM2, UGT1A10 (includes EG:54575), VHL | 18 | 12 | Cell Cycle, Cellular Development, Gene Expression |
| APLNR, BSG, C3AR1, CCRL1, CHEMOKINE, CNR2, CRHR2, CXCR6 (includes EG:10663), EMR1, ERK, Focal adhesion kinase, G protein alphai, Gpcr, GPR21, GPR77, GPR109A, GPRC5A, HRH2, HTR2B, IKK (complex), IL12 (family), Mapk, MC3R, Mmp, PIK3CG, Pka, Pkc(s), PTGER2, RAB32, Rac, RAC2, Ras, S1PR4, SRC, STAT | 8 | 6 | Cell Signaling, Nutritional Disease, Psychological Disorders |
| BRD2, SLAMF9 | 2 | 1 | Cancer, Immunological Disease, Cell Death |
| ARL11, TRIP13 | 2 | 1 | Cell Cycle, Reproductive System Development and Function, Cellular Development |
| CYP4V, CYP4V2 | 2 | 1 |  |
| N-acylglucosamine 2-epimerase, RENBP, ZBED1 | 2 | 1 | Carbohydrate Metabolism, Small Molecule Biochemistry, Lipid Metabolism |
| C2ORF18, HMGCL, MIR93 (includes EG:407050), MS4A7 | 2 | 1 | Lipid Metabolism, Small Molecule Biochemistry, Nucleic Acid Metabolism |
| MIR224 (includes EG:407009), MIR342 (includes EG:100313980), SLC6A3, SYNGR1 | 2 | 1 | Nervous System Development and Function, Drug Metabolism, Small Molecule Biochemistry |
